# Genotype-Environment Interaction Shapes the Microbial Assemblage in Grapevine’s Phyllosphere and Carposphere: An NGS Approach

**DOI:** 10.3390/microorganisms6040096

**Published:** 2018-09-21

**Authors:** Prashant Singh, Sylvain Santoni, Patrice This, Jean-Pierre Péros

**Affiliations:** AGAP, Univ Montpellier, CIRAD, INRA, Montpellier SupAgro, 34000 Montpellier, France; sylvain.santoni@inra.fr (S.S.), patrice.this@inra.fr (P.T.), jean-pierre.peros@inra.fr (J.-P.P.)

**Keywords:** agro-climate zones, genotype, grapevine, microbiome, phyllosphere, PMCs, terroir

## Abstract

Plant surface or phyllosphere is the habitat of hyperdiverse microbial communities and it is always exposed to the fluctuating environmental factors, which is thought to be one of the potential drivers of microbial community structuring. Impact of grapevine genotypes in variable environmental factors (i.e., at different geographic locations) on the phyllosphere has never been studied and is the main objective of this report. Using high throughput short amplicon sequencing of 16S rRNA genes and internal transcribed spacer (ITS), we analyzed the impacts of genotypes of *Vitis Vinifera* (coming from three genetic pool), on the microbial (bacterial and fungal) assemblage in the phyllosphere. First, we performed the analysis of the phyllosphere microbiome while using fifteen genotypes that were chosen to maximize intra-specific diversity and grown in two Mediterranean vineyards. Then, the same analysis was performed on five commercially important varieties of *Vitis vinifera* that were sampled from three different French agro-climatic zones (or terroir: a combination of climate, soils, and human practices). Our study revealed that, at a particular geographic location, genotypes have an impact on microbial assemblage in the phyllosphere and carposphere of leaf and fruit (or berries), respectively, which is more prominent on the carposphere but the effect of terroir was much stronger than the genotype when the leaf phyllosphere of five grapevine varieties grown in different agro-climatic zones was compared. Impacts of the season and exterior plant organs (leaf and berries) on microbial taxa structuring in the phyllosphere was also assessed and presented in this report.

## 1. Introduction

The phyllosphere consists of the aerial parts of the plant and it is one of the most prevalent microbial habitats on earth [1]. Its heterogeneous environment harbors a myriad of microorganisms, like yeast, bacteria, and filamentous fungi and many uncultured organisms [1,2]. The phyllosphere or carposphere microbial communities (PMCs) live at the plant-climate interface and its ability to establish, thrive and reproduce on the leaf or fruit surface depends on several microbial functional traits, such as the ability to attach to the cuticle and to use the foliar nutrients, as well as to the prevailing climatic conditions, like temperature, air humidity, and rain [3,4,5]. Leaf or fruit chemistry, physiology, and morphological structure differ among plant genotype or species, and as all of these traits have a genetic basis, these variations may lead to a different combination of PMCs among plant genotypes [6,7].

The plant genotype may exert selection pressure on PMCs, as often reported in A. thaliana [7,8]. In the literature, impacts of climatic stressors have received much more attention, especially on soil communities than on the PMCs. Nevertheless, phyllosphere faces constant direct exposure to the outside conditions and available pieces of evidence suggest that PMCs significantly alters in response to the climatic stressors like heat, rain or drought [9,10,11,12]. Air pollutants (e.g., oxides of nitrogen and sulfur and particulate matters) that are produced by human activities can alter foliar traits, including cuticle properties [13], leaf chemistry, and phenology [14,15] may also affect the structure of PMCs. Moreover, some of the pollutants can be used as a carbon source by PMCs [16].

The PMCs that are associated with *Vitis vinifera* L., the major crop for fruit and wine production in the world, is less extensively studied when compared to the other habitats (e.g., soil, rhizosphere, and endosphere), especially in relation with the genotypes and the variable climatic conditions or geographic locations. One study suggested that the leaf PMCs are minimally affected by the chemical and biological treatments tested on the plant, but mainly differed according to the grapevine location [17,18]. Berry surfaces also exhibit a huge bacterial and fungal diversity and that can have an impact on grapevine health and wine qualities [19].

In this study, we assessed both the effect of grapevine genotype and environmental factors on the diversity and structure of phyllosphere and carposphere microbiome. When considering that the PMCs on leaf and berry surface plays a crucial role in plant health and fitness as it can modulate leaf or fruit susceptibility to infection [19,20,21], this study could bring new insights to develop innovative and natural biocontrol methods or phytostimulators against grapevine pathogens or rethink breeding schemes for the creation of innovative resistant varieties.

## 2. Materials and Methods

### 2.1. Sample Preparation for PMCs and DNA Extraction

Samples were collected in two sets. In Set1, A total of 279 grapevine cultivars was grown in two vineyards, Chapitre (Supagro field station, Villeneuve-les-Maguelone, Hérault, France) and Vassal (INRA Experimental Unit, Marseillan-Plage, France) near Montpellier (French Mediterranean region). A panel of cultivars representing three genetic pools (western Europe, WW; from eastern Europe, WE; and table grape, TE) was constructed for genome-wide association studies while minimizing relatedness and retaining the main founders of modern cultivated grapevine to optimize the genetic diversity [22]. Five cultivars from each genetic pool, which are far apart based on their distances on PCoA map shown by Nicolas et al. 2016 [22], were selected (Table 1) to maximize the distance between genetic pools. Leaf or berry samples were taken from four to five plants of each cultivar at Spring season (mid of May 2017, before spraying of the fungicides) and harvesting season of (September 2017). Berries were also collected from eleven of these varieties during the harvest season.

In Set2, leaf samples from five commercially important varieties (Cabernet Sauvignon, Chardonnay, Syrah, Grenache, Sauvignon Blanc) were taken from three different geographic locations, (INRA field stations from Bordeaux, Montpellier, and Colmar) within France, representing the three agro-climate zones (Oceanic, Mediterranean, and Continental) of France or different terroirs at the mid of spring season (before spraying of fungicides).

All the samples from both of the sets were washed with an isotonic solution of sodium chloride (0.15 M) containing 0.01% Tween 20 in 50 mL propylene tubes (2–3 leaves and 50–80 g of berries were washed per tube) while using a horizontal shaker for 1h at 100 RPM. Afterward, samples were given an ultrasonic bath for 7–10 min while using Ultrasonic Cleaner (Branson 5510, Marshall Scientific, Hampton, NH, USA) for maximum recovery of microbes from the sample surface. The remaining solution was centrifuged at 4000×g and microbial pellets containing PMCs were transferred in a 2 mL Eppendorf tube and were collected and stored at −20 °C. PMCs from two of these tubes were mixed to make one biological replicate of a single variety and a total of three biological replicates were made for each variety per vineyard. DNA was extracted from each sample by using the ZymoBiomics DNA MicroPrep Kit (Zymo Research, Irvine, CA, USA) following the manufacturer’s instructions.

### 2.2. DNA Amplification and Amplicon Sequence Library Preparation

To access bacterial communities, the V4 region of the 16S ribosomal gene was amplified using primers 515F and 806R and fungal community diversity and abundance were accessed using modified ITS9 and ITS4 primers targeting the ITS2 region [23,24]. Two-step PCR was performed to prepare sequencing libraries. PCR1 was designed to perform amplification of the target regions and to add Illumina Nextera transposase sequence to the amplicons. Both forward and reverse primers for PCR1 were amended with frameshift (FS) sequences in their 5′ overhang to improve sequence diversity and overall read quality [25]. PCR1 was performed in 25 µL reactions with 30 ng of sample DNA while using the KAPA HiFi HotStart (KAPA Biosystems, Wilmington, MA, USA) PCR mix (Initial denaturing at 95 °C followed by 30 cycles of denaturing at 95 °C for 30 s, primer annealing at 57 °C for 60 s, and primer extension at 68 °C for 60 s). Amplicons were purified while using Agencourt AMPure XP beads (Beckman Coulter, Brea, CA, USA) at a bead-to-DNA ratio of 0.7:1, resuspended in 30 μL MilliQ water, and evaluated in agarose gels. In PCR2, Primers from Illumina kit for dual indexing of the amplicons was used. Each cleaned PCR1 product within the same sample received a unique combination of forward and reverse primers (respectively, N7 and S5 Illumina dual index oligos). Afterward, samples were again cleaned while using AmPure XP magnetic beads, pooled in equimolar concentrations, and sequenced using 2×250 bp MiSeq v2 sequencing (Illumina Inc., San Diego, CA, USA).

### 2.3. Data Processing and Analysis

Demultiplexed RAW data files covering all of the samples were imported into the R-environment, (R Core Team, Vienna, Austria). The entire amplicon sequences data was uploaded to the institutional server (http://agap-ng6.supagro.inra.fr/inra). Paired forward and reverse reads from raw data files were trimmed (primer removal) and filtered (base quality) while using the fastqPairedFilter function of the *dada2* package [26] and bases with low-quality scores (<11) were discarded. These filtered files were then processed using Divisive Amplicon Denoising Algorithm (DADA) pipeline which included the steps of dereplication, core denoising algorithm (that models and corrects Illumina-sequenced amplicon errors) and the merging of the base pairs. Merging function provided global ends-free alignment between paired forward and reverse reads and merged them together if they overlapped exactly and a table for amplicon sequence variants (ASVs, a higher analog of operational taxonomic units—OTUs) was constructed. It records the number of times each amplicon sequence variant is observed in each sample. DADA infers sample sequences exactly and resolves differences of as little as one nucleotide [26]. Chimeras were removed using the removeBimeraDenovo function of the same *dada2* package (Table 2 represents the total number of reads available during these steps). ASVs or OTU sequences were assigned a taxonomy using the RDP classifier [27,28] with k-mer size 8 and 100 bootstrap replicates. Afterward, a phyloseq data object was created using phyloseq function of the *phyloseq* package in R [29]. Unassigned taxa and singletons were removed and this data object was then used to calculate microbial abundances, α, β diversity analysis and for other statistical tests using various functions in the *phyloseq* and *vegan* packages [29,30].

Estimates of observed α-diversity [31] were measured within sample categories using estimate_richness function of the *phyloseq* package. Relative abundances of microbial genera and phylum were plotted using the *ggplot2* package [32] after transforming abundance data into relative abundances. Multidimensional scaling (MDS, also known as principal coordinate analysis; PCoA) was performed while using the Bray-Curtis dissimilarity matrix between samples and visualized by using their base functions in the *phyloseq* package.

### 2.4. Statistical Analysis

We analyzed all of the amplicon sequences in R version 3.3.4 using above mentioned Bioconductor packages. CRAN packages *plyr* and *ggplot2* [32,33] were also used to draw the figures. We assessed the statistical significance (*p* < 0.05) throughout and whenever necessary, we adjusted *p*-values for multiple comparisons according to the Benjamini and Hochberg method to control False Discovery Rate [34], while performing multiple testing on taxa abundance according to sample categories. We performed an analysis of variance or ANOVA [35] among sample categories while measuring the *Observed* estimates of α-diversity (richness of unique OTUs). Stratified permutational multivariate analysis of variance (PERMANOVA) with 999 permutations was conducted on all principal coordinates that were obtained during PCoA with the adonis function of the *vegan* package, to observe the statistical significance of clusters according to the sample categories.

## 3. Results

16S and ITS Amplicon sequencing of all the samples from both sets gave millions of reads. Table 2 describes the total number of reads that were obtained during the processing steps. 30–35% of the reads were trimmed due to the filtering parameters and chimera removal in both 16S and ITS datasets.

A total of 13521 + 4581 bacterial and 10162 + 3164 fungal OTUs were recovered from 213 + 45 samples of both sets (Table 2) and after phylum level assignment 9516 + 3755 bacterial and 6749 + 1800 fungal OTUs were retained and used for further analysis.

### 3.1. Seasonal Shifts in Leaf Microbiome Structure

**Figure 1:**
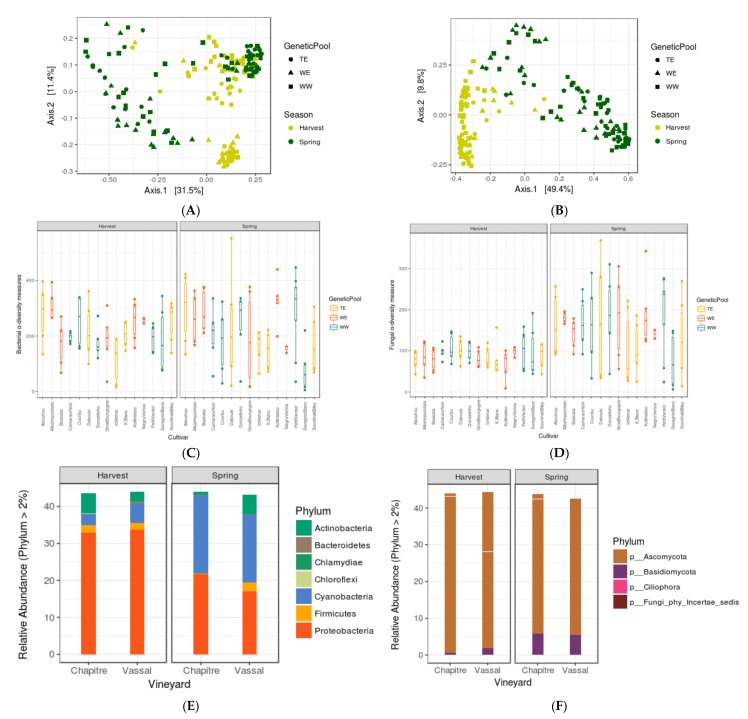
PCoA analysis on leaf data (**A**,**B**); PCoA plots representing the compositional dissimilarity in leaf communities (both axis covered >40% of the variation) and *Observed* (**C**) bacterial and (**D**) fungal α-diversity measures of each variety (X-axis) grouped in two season and relative abundances of (**E**) bacterial and (**F**) fungal Phylum during spring and harvest season. *n* = 180.

PCoA analysis on leaf data (from spring and harvest season) showed fluctuation in taxonomic structuring (Figure 1A,B) between two seasons (PERMANOVA for 16S data: at *F* = 5.285, *p* < 0.001; for ITS data: at *F* = 99.057, *p* < 0.001), but the *Observed* α-diversity estimates (Figure 1C, for bacterial data) indicated that the richness for unique bacterial OTUs did not change between seasons (ANOVA, at *F* = 2.973, *p* > 0.085). On the contrary, *Observed* α-diversity estimates for fungal data (Figure 1D) displayed significant differences in richness of unique fungal OTUs (ANOVA, at *F* = 47.958, *p* < 1.2 × 10^−10^). In combination, our results indicated a compositional dissimilarity for bacterial populations between two seasons, but the uniqueness of the composition (or bacterial diversity) did not change, which was further confirmed by the relative abundance analysis (Figure 1E). From spring to harvest season, leaf microbiota loose significant amount of Cyanobacteria (79.5%) and gained an ample amount of Proteobacteria (28%), which was probably the cause of the seasonal drift obtained. On the other hand, there was a strong impact of season on fungal composition as well as diversity (Figure 1F) in the phyllosphere, which was more evident at the genus or species level (Supplementary Figure S1).

### 3.2. Assessing the Impacts of Grapevine Cultivars and Genetic Pools

**Figure 2:**
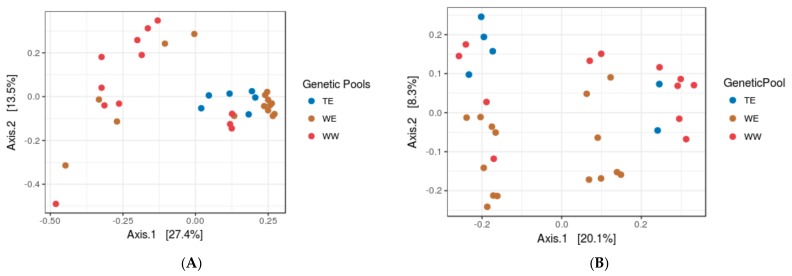
PCoA on (**A**) bacterial and (**B**) fungal microbiome data of berry displaying the impact of genetic pools on taxa structuring on the surface (both axis covered >25% of the variation in data). *n* = 33.

By PCoA analysis on leaf microbiome data over two seasons, we did not observe genetic pool wise variation (Figure 1A,B Shape represent genetic pools) on taxonomic structuring (PERMANOVA, at *F* = 2.018, *p* = 0.083) in the phyllosphere. However, at each individual season, we observed some significant differences in α-diversity measures (Figure 1C,D) and PCoA clusters, according to grapevine cultivars and genetic pools (Table 3). On the other hand, PCoA analysis of berry microbiome data displayed stronger effects (Figure 2A,B) of both the factors (Table 4) on PMC structuring.

### 3.3. Impact of Organs

**Figure 3:**
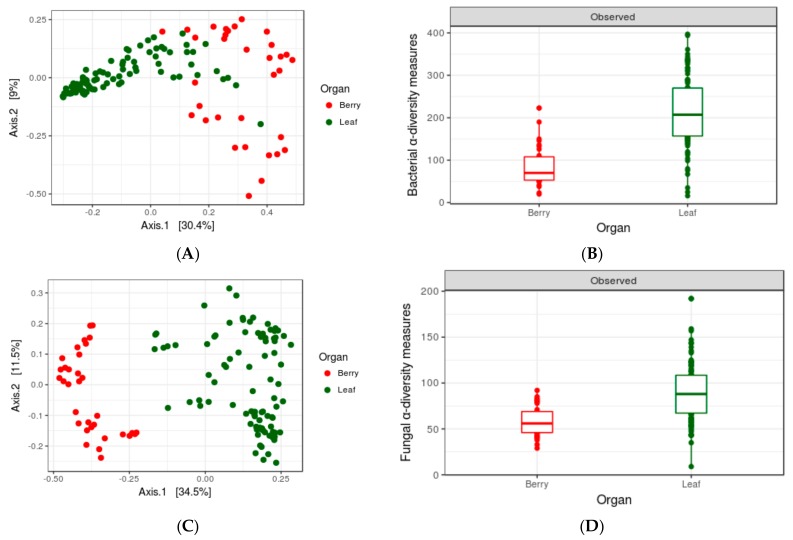
PCoA plot representing compositional dissimilarity for (**A**) bacterial and (**B**) fungal population between leaf and berry samples (both axis covered ~40% of the variation) and *Observed* α-diversity measures for (**C**) bacteria and (**D**) fungi for two organs. *n* = 123.

Comparisons of PMCs on leaves and berries (samples from Set1, collected at harvest season, Figure 3 revealed a very clear differentiation of microbiome communities on both organs. PCoA revealed a clear difference in taxonomic structuring (Figure 3A,B; PERMANOVA for 16S data: *F* = 14.6, *p* = 0.001; for ITS data: *F* = 45.738, *p* = 0.001), while the α-diversity estimates displayed very significant differences in OTU richness (Figure 3C,D) between the leaves and berries (ANOVA for 16S data: *F* = 7.17, *p* =6.95 × 10^−14^; for ITS data: *F* = 4.575, *p* = 0.000143), multiple testing on taxa abundance between the two organs revealed 20 bacterial and 26 fungal genera, differentially abundant (Supplementary Table S1).

### 3.4. Impact of Agro-Climate Zones (or Terroir) and Genotype

**Figure 4:**
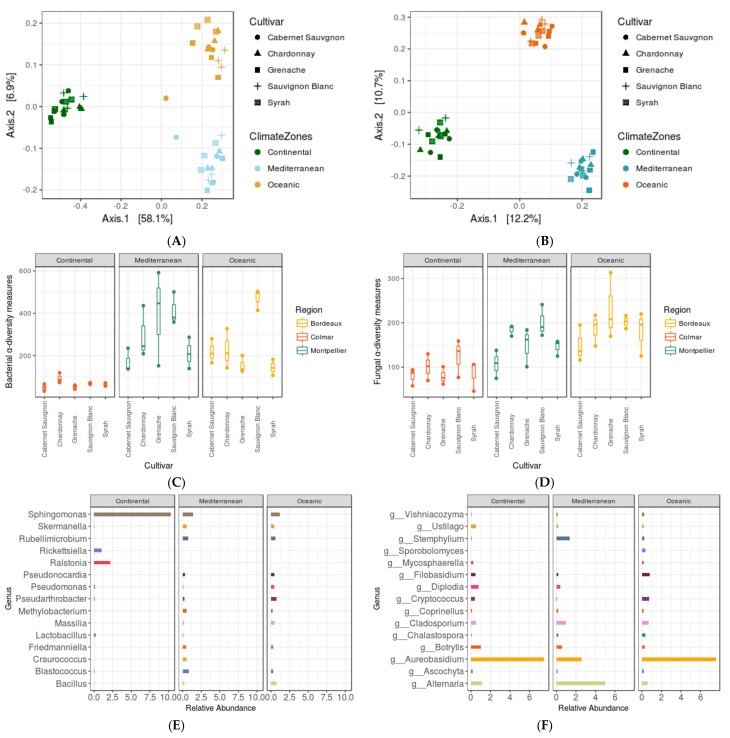
Set2 microbiome data. PCoA plots displaying strong (**A**) bacterial and (**B**) fungal compositional dissimilarity among agro-climate zones and *Observed* (**C**) bacterial and (**D**) fungal α-diversity measures of each variety (X-axis) grouped in three agro-climate zones and relative abundance plot for (**E**) bacterial and (**F**) fungal genera displaying differential abundance of few genera among three agro-climate zones (or region). *n* = 45.

Analysis of the microbiome of leaf phyllosphere on the 5 grapevine cultivars of set2 in the three very diverse French regions revealed a strong effect of terroir. A very clear differentiation of the samples collected in the three regions was observed on PCoA plots for bacterial (Figure 4A,B). Leaf PMCs for the five cultivars indeed clustered only according to grapevine locations (PERMANOVA for 16S data: *F* = 12.98, *p* = 0.001; for ITS data: *F* = 6.094, *p* = 0.001). The α-diversity estimates also indicated very significant differences in OTU richness (Figure 4C,D) between the three regions (ANOVA for 16S data: *F* = 25.73, *p* = 3.11 × 10^−7^; for ITS data: at *F* = 26.329, *p* = 2.5 × 10^−7^). In combination, these results illustrated that French agro-climatic zones have very strong impacts in shaping the microbial assembly in the leaf phyllosphere. In addition, it has also suggested that there is not only a region-wise difference in taxonomic compositions, but each region (or agro-climate zone) has a unique microbial signature (Figure 4E,F). Multiple testing (with corrected *p*-values to control false discovery rates) on taxa abundance gave 31 bacterial and 21 fungal genera, which were differentially abundant among these three regions representing different environment (Supplementary Table S2).

A lower but significant cultivar level differences on Observed α-diversity estimates (ANOVA for 16S data: *F* = 7.18, *p* = 0.00022; for ITS data: *F* = 3.798, *p* = 0.013) was however also observed (Figure 4C,D). Within a specified region, genotype had also an effect on the diversity in both microbial and fungal communities even if PCoA analysis did not reveal any differentiation according to the cultivar (PERMANOVA for 16S data: *F* = 0.893, *p* = 0.675; for ITS data: *F* = 1.171, *p* = 0.851).

## 4. Discussion

Phyllosphere of the grapevines is quite a neglected milieu and many questions related to this microbial habitat are still unanswered, especially the relative impacts of potential factors that could play key roles in shaping the microbial community structure in the phyllosphere. A better understanding of the principal factors affecting community structure and multitrophic interactions in the phyllosphere will be the key to develop new strategies for grapevine protection. The better we understand the role of these stressors and PMCs that they affect, the better we would be able to predict and protect grapevine against pathogen infection.

In this study, we first explored the microbial communities present in the Mediterranean, Continental and Oceanic vineyards. Major bacterial and fungal taxa (at genus level) were *Pseudomonas*, *Sphingomonas*, *Pantoea, Skermanella* & *Aureobasidium*, *Filobasidium*, *Alternaria*, and *Stemphylium*, respectively. Differences in relative abundances of major taxa were quite visible according to agro-climate zones (or growing region) as compared to cultivars (grouped in three genetic pool), growing in the Mediterranean (Supplementary Figures S2 and S3). We mainly investigated the impacts of grapevine genotypes (or cultivars) and of terroir on the assemblage of PMCs using a culture-independent method. In the Mediterranean vineyards, grapevine cultivars, and their genetic pools had a significant impact on leaf and berry microbiome and the impact is stronger on the berry surface. Assuming that the PMCs on berries would also be present on wine must this result is in line with reports, suggesting that the microbiota exhibits varietal level differences in wine musts of Chardonnay and Cabernet Sauvignon [36,37].

While comparing the impacts of climatic stressors and cultivars at three different locations, we observed a very strong impact of French agro-climate zones or terroirs. Although the impact of genetic factors was significant but much lower in comparison with terroir, which suggests that genotype-by-environment interactions contributed to the complexity of microbiome assembly. Such interactions also represent the cumulative influence of a potentially large number of environmental factors can be involved: soil type, for example, was different in the locations tested. Since the epiphytes (PMCs) that are associated with grapevine could originate from soil [38], leaf communities could be influenced by soil chemistry or other abiotic factors of the regions where plants are grown, leading to these region-specific unique microbial signatures.

Few strains of Sphingomonas, which was found quite abundant in all three regions (Figure 4E) were recently reported in plant protection against a bacterial pathogen (*P. syringae* DC3000) in *A. thaliana* model system [39]. Although, the molecular basis of pathogen reduction is unknown, but available evidence suggests that several traits contribute to the outcome of plant protection [40]. Differential abundance of Sphingomonas in grapevine grown in different regions should thus be studied in future in relation to plant traits to assess its impacts on grapevine health. Similarly, a fungal genera *Aureobasidium* was also quite abundant in all three regions (Figure 4F) and this prevalence of *Aureobasidium* was due to the presence of *A. pullulans* (relative abundance >12%, Supplementary Figure S1). *A. pullulans* have an antagonistic activity for Botrytis molds and for certain bacteria like Bacillus [28,41], which probably explains the lower prevalence of Bacillus and Botrytis in our data (Figure 4E,F).

Seasonal shifts in phyllosphere microbiome structure and the impacts of plant organs were also observed. At a particular location, the microbial composition of few bacterial phyla might change while bacterial diversity does not change during season shift. For example, cyanobacteria (photosynthetic bacteria) change its abundance from high to lower due to season change from spring to harvest. Lower daylight presence in harvest season probably explains these changes. These results are coherent with another grapevine (Tempranillo) related study [38]. On contrary, fungal community diversity and their relative abundances, both were significantly impacted by season. Apart from genotype and terroir, the vineyard management practices could also be the possible reason for these differences [9]. Although a significant fraction of the members of PMCs were shared between plant organs (leaves and berries), we observed distinct assemblage patterns between both organs, which is also in accordance with recently published reports [25,37]. These differences among organs do not only reflect the compositional differences (or difference in the relative abundance of shared OTUs), but also the diversity in taxa present.

## 5. Conclusions

Our present study assessed the major microbial diversity present over French agro-climate zones and compared the many facets of factors that may influence the microbiome structure in the phyllosphere, with special focus on relative selection pressure that is exerted by grapevine genotype and its interaction with different climatic conditions (or terroir represented by French agro-climate zones), which may improve our chances to find genes that control PMCs on phyllosphere, and simultaneously increase our confidence that those genes are actually important in realistic environments, and probably those genes would give us new insights for breeding new and healthy grape varieties displaying better traits.

## Figures and Tables

**Table 1:** Schematic representation of 15 grapevine cultivars (grouped in three genetic pools) that were sampled in Set1.

		Genetic Pools	
	WW	WE	TE
	Donzelinho	Basicata	Ichkimar
**Cultivars of *Vitis Vinifera***	Petit Verdot	Negru Vertos	Khoussaïné blanc
	Camaraou Noir	Alba Imputotato	Sourkhak Biley
	Courbu	Gros Bourgogne	Abouhu
	Savagnin Blanc	Koilliniatico	Dabouki

**Table 2:** Total number of reads during each step of microbiome data (16S/ITS) analysis.

Data	Number of Samples	Input Reads	Filtered Reads	Denoised and Merged	OTUs
**16S data**					
Set1	213	16113978	10874688	7795650	13521
Set2	45	7460569	5294234	3866297	4581
**ITS data**					
Set1	213	14780926	13600570	9900482	10162
Set2	45	6683219	4564572	2450315	3164

**Table 3:** Factors predicting the impacts of grapevine varieties and genetic pools on the leaf bacterial communities at each season.

	Spring		Harvest	
Factors	ANOVA (on α-Diversity Measures)	PERMANOVA on PCoA Clusters	ANOVA (on α-Diversity Measures)	PERMANOVA on PCoA Clusters
**16S data**				
Cultivars	*F* = 2.361, *p* = 0.0009	*F* = 1.129, *p* = 0.002	*F* = 2.837, *p* = 0.002	*F* = 2.737, *p* = 0.001
Genetic Pool	*F* = 1.54, *p* = 0.221	*F* = 1.178, *p* = 0.082	*F* = 1.189, *p* = 0.308	*F* = 2.617, *p* = 0.001
**ITS data**				
Cultivars	*F* = 1.17, *p* = 0.315	*F* = 1.583, *p* = 0.006	*F* = 0.752, *p* = 0.715	*F* = 2.098, *p* = 0.001
Genetic Pool	*F* = 1.384, *p* = 0.255	*F* = 2.218, *p* = 0.015	*F* = 3.368, *p* = 0.038	*F* = 2.764, *p* = 0.001

**Table 4:** Factors predicting the impacts of grapevine varieties and genetic pools on bacterial communities on berry surface at Harvest season.

Factors	ANOVA (on α-Diversity Measures)	PERMANOVA on PCoA Clusters
**16S data**		
Cultivars	*F* = 2.546, *p* = 0.002	*F* = 2.598, *p* = 0.001
Genetic Pool	*F* = 4.261, *p* = 0.023	*F* = 4.612, *p* = 0.001
**ITS data**		
Cultivars	*F* = 4.575, *p* = 0.00142	*F* = 3.169, *p* = 0.001
Genetic Pool	*F* = 2.739, *p* = 0.07	*F* = 4.612, *p* = 0.003

## Supplementary Materials

The following are available online at http://www.mdpi.com/2076-2607/6/4/96/s1, Table S1a: Differentially abundant bacterial genera between Leaf and Berries, Table S1b: Differentially abundant fungal genera between Leaf and Berries, Table S2a: Differentially abundant bacterial genera among three climate zones, Table S2b: Differentially abundant fungal genera among three climate zones, Figure S1: Relative Abundance major species between two seasons, displaying the uniqueness of the fungal microbiome structure at Spring and Harvest season, Figure S2: Relative abundances of major (A) bacterial and (B) fungal taxa (top 25, at genus level) of each cultivar (grouped in three genetic pools). Set1. *n* = 213, Figure S3: Relative abundances of major (A) bacterial and (B) fungal taxa (top 25, at genus level) of each cultivar (grouped in three geographic locations). Set2. *n* = 45.
