# Supplementary material for: Genotype-Environment Interaction Shapes the Microbial Assemblage in Grapevine’s Phyllosphere and Carposphere: An NGS Approach
Source: Microorganisms. 2018 Sep 21;6(4):96. doi: 10.3390/microorganisms6040096 (PMC6313654; doi:10.3390/microorganisms6040096)
Supplement: Supplementary file 1 [file microorganisms-06-00096-s001.pdf]

**Supplementary Table S1a:** Differentially abundant bacterial genera between Leaf and Berries

| OTUs | Genus | Corrected P-values | FDRs |
|---|---|---|---|
| **OTU94** | *Geodermatophilus* | 0.0060 | 0.00066 |
| **OTU428** | *Kineococcus* | 0.0060 | 0.00066 |
| **OTU903** | *Hymenobacter* | 0.0060 | 0.00066 |
| **OTU152** | *Arthrobacter* | 0.0060 | 0.00066 |
| **OTU39** | *Paenisporosarcina* | 0.0060 | 0.00066 |
| **OTU288** | *Paenibacillus* | 0.0060 | 0.00066 |
| **OTU101** | *Microbacterium* | 0.0060 | 0.00066 |
| **OTU460** | *Planifilum* | 0.0060 | 0.00066 |
| **OTU200** | *Methylobacterium* | 0.0060 | 0.00066 |
| **OTU72** | *Cutibacterium* | 0.0060 | 0.00066 |
| **OTU134** | *Nocardioides* | 0.0060 | 0.00066 |
| **OTU415** | *Noviherbaspirillum* | 0.0060 | 0.00066 |
| **OTU56** | *Massilia* | 0.0060 | 0.00066 |
| **OTU374** | *Adhaeribacter* | 0.0060 | 0.00066 |
| **OTU26** | *Gluconobacter* | 0.0060 | 0.00066 |
| **OTU451** | *Roseomonas* | 0.0117 | 0.00123 |
| **OTU897** | *Streptococcus* | 0.0172 | 0.00174 |
| **OTU193** | *Domibacillus* | 0.0278 | 0.00275 |
| **OTU849** | *Tumebacillus* | 0.0329 | 0.00312 |
| **OTU211** | *Actinomycetospora* | 0.0426 | 0.00396 |

**Supplementary Table S1b:** Differentially abundant fungal genera between Leaf and Berries

| OTUs | Genus | Species | Corrected P-values | FDRs |
|---|---|---|---|---|
| OTU2 | *g__Alternaria* | *s__alternata* | 0.0021 | 0.0003764706 |
| OTU4 | *g__Cladosporium* | *s__delicatulum* | 0.0021 | 0.0003764706 |
| OTU33 | *g__Sporobolomyces* | *s__roseus* | 0.0021 | 0.0003764706 |
| OTU18 | *g__Sporobolomyces* | *s__roseus* | 0.0021 | 0.0003764706 |
| OTU62 | *g__Aureobasidium* | *s__pullulans* | 0.0021 | 0.0003764706 |
| OTU49 | *g__Rhodotorula* | *s__babjevae* | 0.0021 | 0.0003764706 |
| OTU11 | *g__Filobasidium* | *s__magnum* | 0.0021 | 0.0003764706 |
| OTU12 | *g__Alternaria* | *s__alternata* | 0.0021 | 0.0003764706 |
| OTU32 | *g__Sporormia* | *NA* | 0.0021 | 0.0003764706 |
| OTU19 | *g__Alternaria* | *s__alternata* | 0.0021 | 0.0003764706 |
| OTU79 | *g__Rhodotorula* | *s__babjevae* | 0.0021 | 0.0003764706 |
| OTU61 | *g__Quambalaria* | *s__cyanescens* | 0.0021 | 0.0003764706 |
| OTU83 | *g__Metschnikowia* | *s__pulcherrima* | 0.0021 | 0.0003764706 |
| OTU73 | *g__Metschnikowia* | *s__pulcherrima* | 0.0021 | 0.0003764706 |
| OTU96 | *g__Metschnikowia* | *s__pulcherrima* | 0.0021 | 0.0003764706 |
| OTU35 | *g__Citeromyces* | *s__matritensis* | 0.0021 | 0.0003764706 |
| OTU70 | *g__Acaromyces* | *s__ingoldii* | 0.0021 | 0.0003764706 |
| OTU5 | *g__Aureobasidium* | *s__pullulans* | 0.0041 | 0.0006736842 |
| OTU97 | *g__Metschnikowia* | *s__pulcherrima* | 0.0041 | 0.0006736842 |
| OTU136 | *g__Cryptococcus* | *s__heimaeyensis* | 0.0123 | 0.00192 |
| OTU71 | *g__Filobasidium* | *s__stepposum* | 0.0154 | 0.0024380952 |
| OTU108 | *g__Metschnikowia* | *s__pulcherrima* | 0.0176 | 0.0026181818 |
| OTU51 | *g__Filobasidium* | *s__magnum* | 0.0187 | 0.0027826087 |
| OTU85 | *g__Saccharomycopsis* | *s__vini* | 0.0243 | 0.0034666667 |
| OTU34 | *g__Botrytis* | *s__caroliniana* | 0.0337 | 0.0044307692 |
| OTU52 | *g__Rhizopus* | *s__arrhizus* | 0.0337 | 0.0044307692 |

**Supplementary Table S2a:** Differentially abundant bacterial genera among three climate zones

| OTUs | Genus | Corrected *P*-values | FDRs |
|---|---|---|---|
| OTU29 | *Modestobacter* | 0.0032 | 0.003833333 |
| OTU87 | *Microvirga* | 0.0032 | 0.003833333 |
| OTU114 | *Rubellimicrobium* | 0.0032 | 0.003833333 |
| OTU66 | *Friedmanniella* | 0.0032 | 0.003833333 |
| OTU161 | *Craurococcus* | 0.0032 | 0.003833333 |
| OTU271 | *Neorhizobium* | 0.0032 | 0.003833333 |
| OTU55 | *Paracoccus* | 0.0063 | 0.0046 |
| OTU50 | *Blastococcus* | 0.0063 | 0.0046 |
| OTU61 | *Kocuria* | 0.0063 | 0.0046 |
| OTU113 | *Rathayibacter* | 0.0063 | 0.0046 |
| OTU1 | *Sphingomonas* | 0.0114 | 0.008363636 |
| OTU218 | *Paenisporosarcina* | 0.0143 | 0.009583333 |
| OTU7 | *Ralstonia* | 0.0246 | 0.012266667 |
| OTU124 | *Kocuria* | 0.0246 | 0.012266667 |
| OTU250 | *Deinococcus* | 0.0246 | 0.012266667 |
| OTU279 | *Devosia* | 0.0269 | 0.0129375 |
| OTU45 | *Methylobacterium* | 0.029 | 0.013529412 |
| OTU11 | *Rickettsiella* | 0.0358 | 0.016611111 |
| OTU14 | *Pseudoarthobacter* | 0.0032 | 0.00328 |
| OTU171 | *Acinetobacter* | 0.0032 | 0.00328 |
| OTU262 | *Chamaesiphon_PCC-7430* | 0.0032 | 0.00328 |
| OTU102 | *Rubellimicrobium* | 0.0032 | 0.003285714 |
| OTU152 | *Pleurocapsa_PCC-7319* | 0.0058 | 0.00575 |
| OTU333 | *Aliterella_CENA595* | 0.0111 | 0.010222222 |
| OTU57 | *Methylobacterium* | 0.0033 | 0.003833333 |
| OTU30 | *Skermanella* | 0.0054 | 0.00575 |
| OTU42 | *1174-901-12* | 0.012 | 0.0115 |
| OTU72 | *Staphylococcus* | 0.012 | 0.0115 |
| OTU95 | *Snodgrassella* | 0.0112 | 0.012545455 |
| OTU29 | *Modestobacter* | 0.019 | 0.013416667 |
| OTU55 | *Paracoccus* | 0.0237 | 0.015923077 |

**Supplementary Table S2b:** Differentially abundant fungal genera among three climate zones

| OTUs | Genus | Corrected P-values | FDRs |
|---|---|---|---|
| OTU30 | *g__Sporobolomyces* | 0.0019 | 0.00158 |
| OTU3 | *g__Alternaria* | 0.0019 | 0.00158 |
| OTU54 | *g__Sporobolomyces* | 0.0019 | 0.00158 |
| OTU34 | *g__Ascochyta* | 0.0019 | 0.00158 |
| OTU9 | *g__Cryptococcus* | 0.0019 | 0.00158 |
| OTU66 | *g__Hyperphyscia* | 0.0084 | 0.005266667 |
| OTU1 | *g__Aureobasidium* | 0.0166 | 0.0079 |
| OTU8 | *g__Stemphylium* | 0.0231 | 0.009875 |
| OTU37 | *g__Filobasidium* | 0.0285 | 0.011411111 |
| OTU61 | *g__Ganoderma* | 0.0313 | 0.01185 |
| OTU9 | *g__Cryptococcus* | 0.0014 | 0.0009875 |
| OTU31 | *g__Stigmina* | 0.0014 | 0.0009875 |
| OTU6 | *g__Cladosporium* | 0.0014 | 0.0009875 |
| OTU73 | *g__Aureobasidium* | 0.0014 | 0.0009875 |
| OTU39 | *g__Symmetrospora* | 0.0047 | 0.002633333 |
| OTU56 | *g__Coprinopsis* | 0.0097 | 0.004253846 |
| OTU62 | *g__Aspergillus* | 0.0138 | 0.004253846 |
| OTU115 | *g__Exophiala* | 0.0138 | 0.004253846 |
| OTU21 | *g_Ustilago* | 0.0165 | 0.005078571 |
| OTU16 | *g__Alternaria* | 0.0159 | 0.0069125 |
| OTU17 | *g__Chalastospora* | 0.0286 | 0.010772727 |

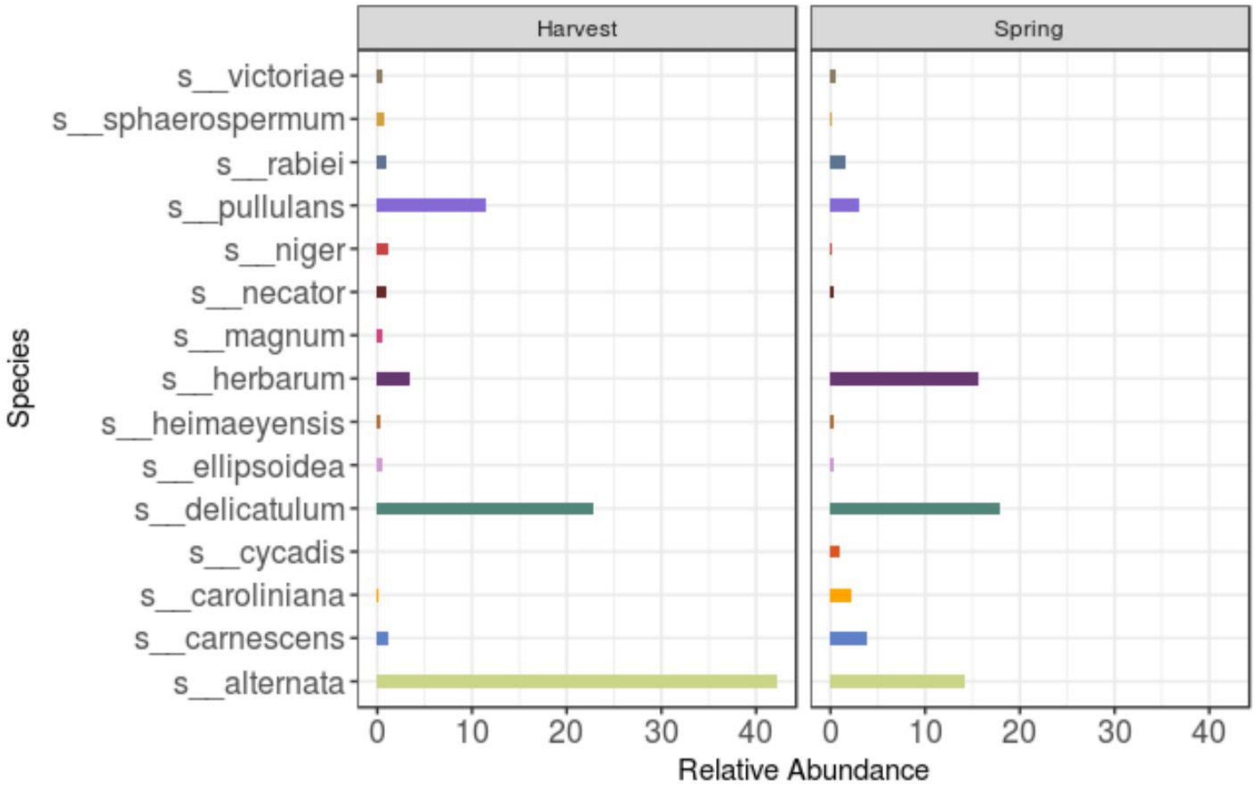


**Supplementary Figure S1.** Relative Abundance major species between two season, displaying the uniqueness of the fungal microbiome structure at Spring and Harvest season.

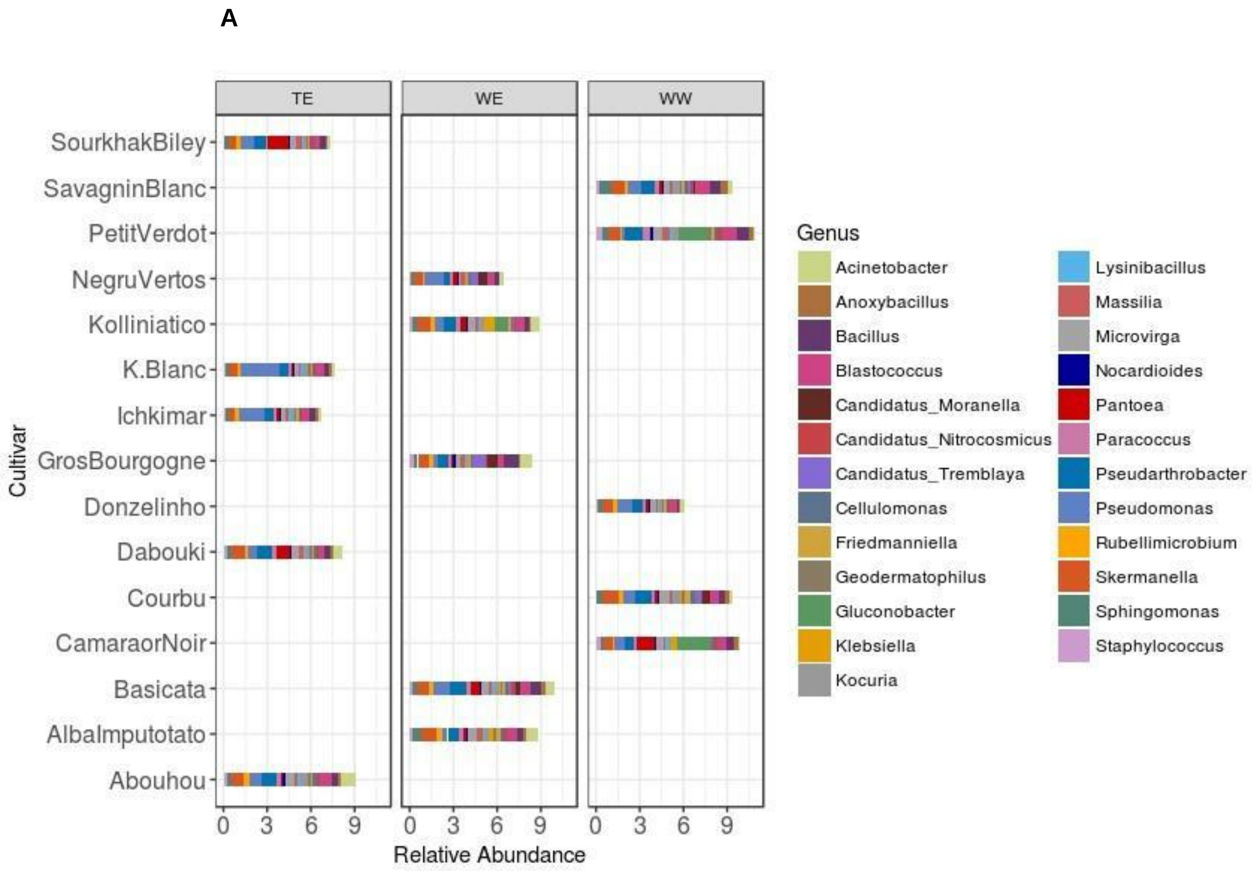


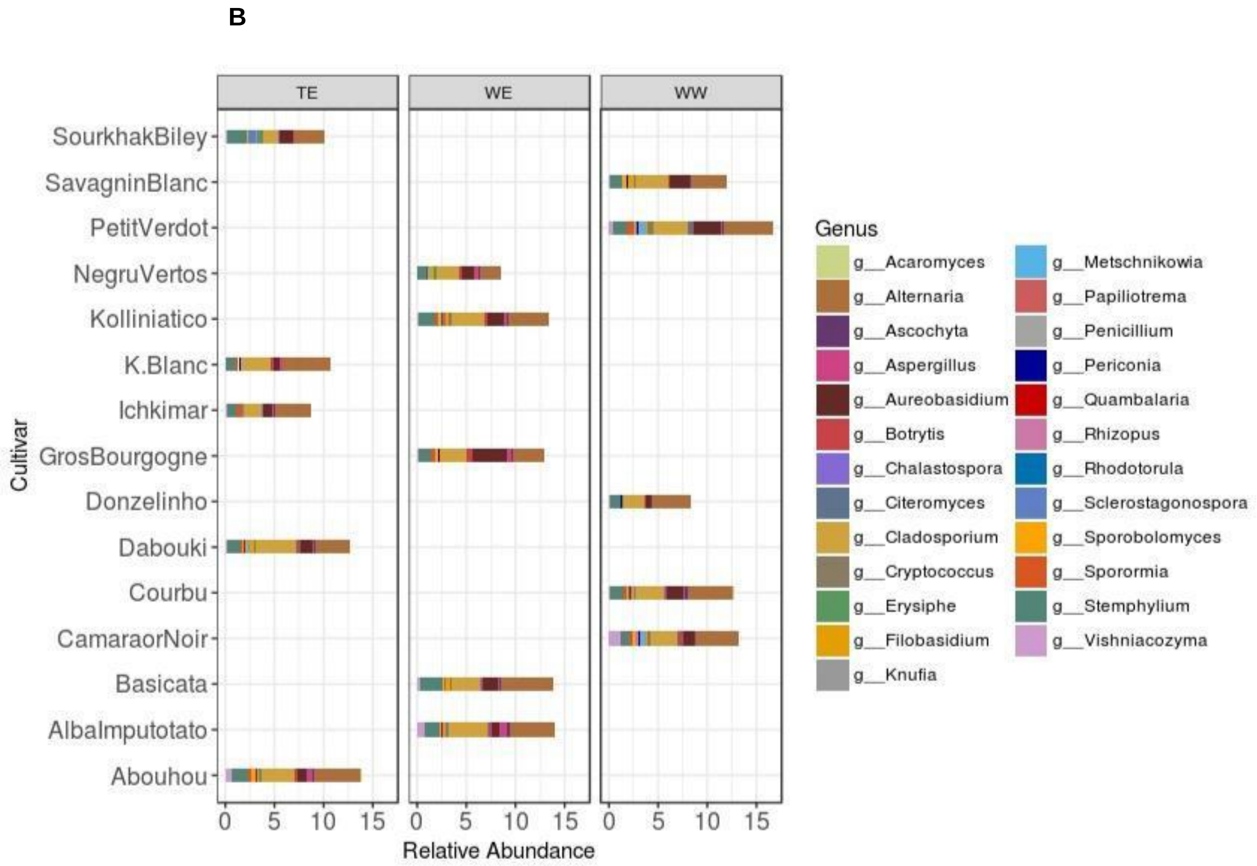


**Supplementary Fig S2.** Relative abundances of major (A) bacterial and (B) fungal taxa (top 25, at genus level) of each cultivar (grouped in three genetic pools). Set1. N = 213.

A

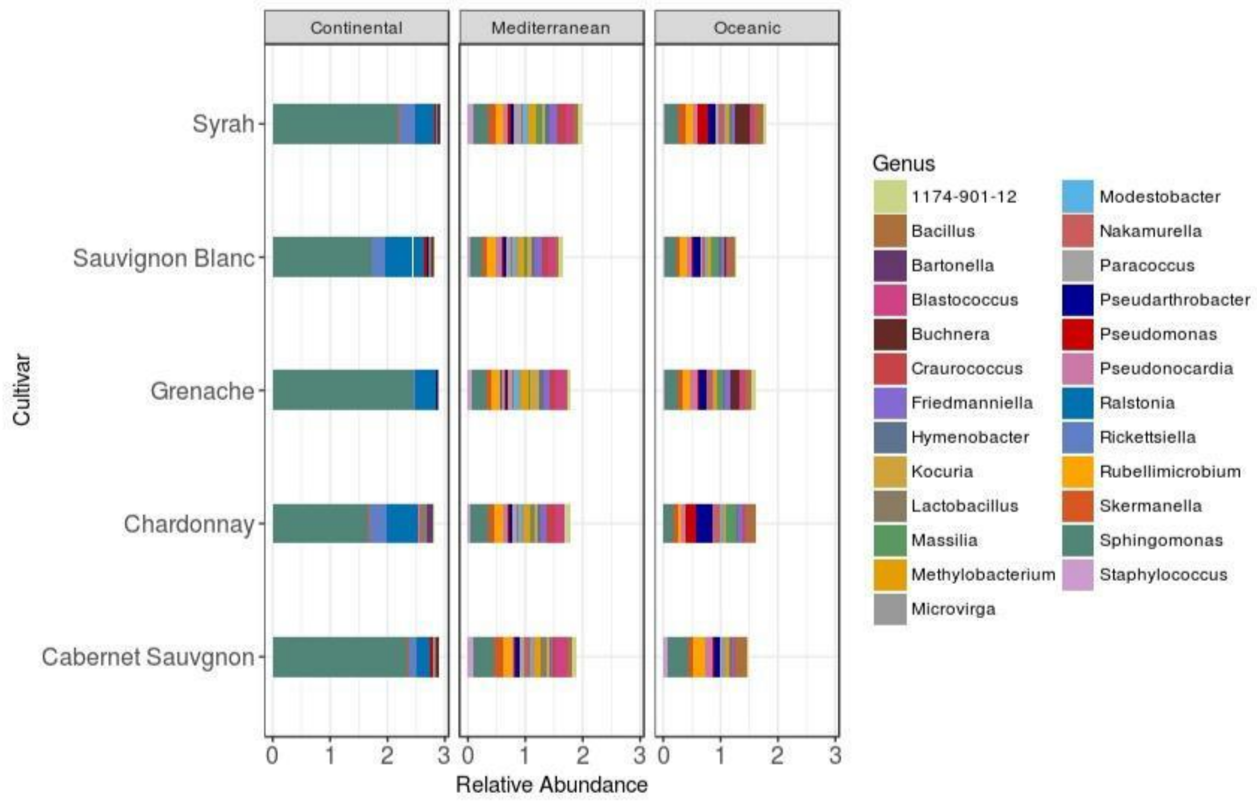


B

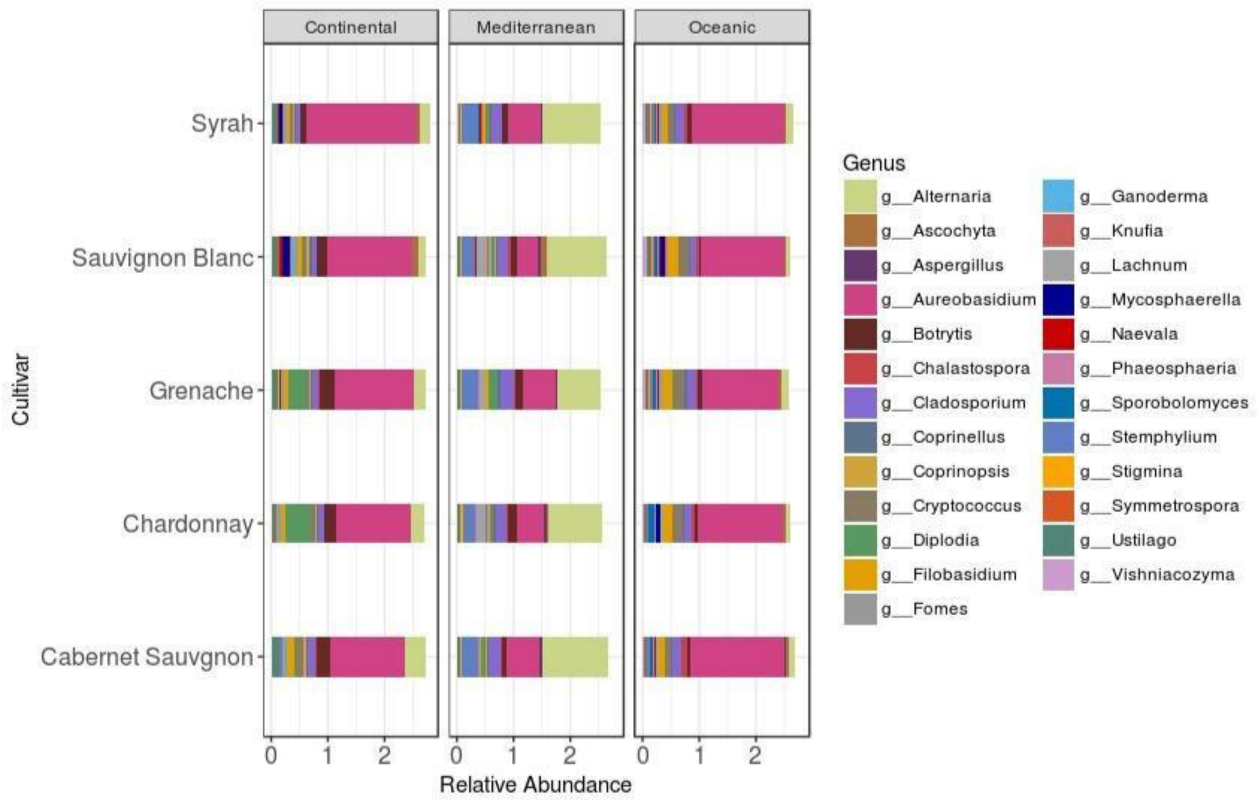


**Supplementary Fig S3.** Relative abundances of major (A) bacterial and (B) fungal taxa (top 25, at genus level) of each cultivar (grouped in three geographic locations). Set2. N = 45.
